# Analysis of clinical efficacy of comprehensive nursing intervention on elderly patients with hypertension combined with hyperuricemia

**DOI:** 10.12669/pjms.39.6.7233

**Published:** 2023

**Authors:** Na Lu, Jing Liu, Pan Cai

**Affiliations:** 1 Na Lu, Department of Internal Medicine Neurology, Baoding No.1 Central Hospital, Baoding 071000, Hebei, China; 2 Jing Liu, Department of Rheumatology and Immunology, Baoding No.1 Central Hospital, Baoding 071000, Hebei, China; 3 Pan Cai, Department of Rheumatology and Immunology, Baoding No.1 Central Hospital, Baoding 071000, Hebei, China

**Keywords:** Comprehensive Nursing Intervention, Elderly Patients, Hyperuricemia, Hypertension

## Abstract

**Objective:**

To evaluate the clinical efficacy of the comprehensive nursing intervention on elderly patients with hypertension combined with hyperuricemia.

**Methods:**

This was a retrospective study. One hundred elderly patients with hypertension combined with hyperuricemia admitted to the Baoding No.1 Central Hospital from May 2019 to May 2020 were included and randomly divided into two groups. Patients in the control group were treated with conventional nursing intervention, while those in the experimental group were treated with comprehensive nursing intervention based on the therapy in the control group. The improvement of compliance behavior, clinical efficacy, quality of life and satisfaction with hypertension combined with hyperuricemia before and after treatment were compared and analyzed between the two groups. The proportion of patients in both groups who developed gout or renal insufficiency was recorded, and their long-term treatment outcomes were compared and analyzed.

**Results:**

After the comprehensive nursing intervention, the number of cases of compliance behaviors in the experimental group was significantly higher than that in the control group(p<0.05). The systolic and diastolic blood pressure and uric acid levels were significantly lower in the experimental group compared to the control group after the intervention(p=0.00). The scores of physical function, psychological function, social function and material life status improved significantly in the experimental group compared to the control group after the intervention(p=0.00). The satisfaction rate of the experimental group was significantly higher than the control group(p=0.02). The proportion of patients in the experimental group who developed gout was significantly lower than that in the control group(p=0.03).

**Conclusion:**

Comprehensive nursing intervention plays a vital role in the treatment and prognosis of hypertension combined with hyperuricemia.

## INTRODUCTION

Uric acid, as the end product of purine base metabolism in nucleic acids, is generally produced from hypoxanthine and xanthine, products of the purine synthesis process, catalyzed by xanthine oxidase.^1^ Excessive blood uric acid exerts its strong oxidative effect and causes damage to the body. Yip et al.^2^,^3^ Concluded that asymptomatic hyperuricemia has a close bearing on a variety of comorbidities, including hypertension, chronic kidney disease, coronary artery disease and diabetes mellitus. Changes in people’s lifestyles have gradually increased the level of blood uric acid. In recent years, hypertension combined with hyperuricemia has become a common condition that makes inroads in the elderly.

Hypertension combined with hyperuricemia is a chronic process that requires increased awareness and compliance in the treatment of elderly patients with this condition.^4^ Comprehensive nursing intervention is a new type of nursing care that is tailored to a particular disease and patient’s condition in a pre-determined way.^5^ A growing body of evidence shows^6^ that comprehensive nursing has advantages in improving patients’ perceptions of care as well as treatment adherence. Clearly, nursing staff plays an important role here. It was found that nursing interventions taken in the treatment of gout and hyperuricemia can play a positive role.^7^ Scientific and rational nursing interventions are an important part of the treatment of gout and hyperuricemia.^8^ With the current rapid development of nursing, comprehensive nursing intervention, an intervention method based on continuum thinking, is also widely used in clinical practice. It boasts many advantages, such as personalized, holistic, and effective.^9^,^10^ In this study, the comprehensive nursing intervention was used in the clinical diagnosis and treatment of elderly patients with hypertension combined with hyperuricemia, and certain effects were obtained.

## METHODS

This was a retrospective study. One hundred elderly patients with hypertension combined with hyperuricemia admitted to the Baoding No.1 Central Hospital from May 2019 to May 2020 was included and randomly divided into two groups: the experimental group and the control group, with 50 cases in each group. No statistically significant difference was observed between the two groups in the comparison of general information (Table-I).

**Table-I:** Comparative analysis of general information between the experimental group and the control group (*χ̅*±*S*) n=50.

Indicators	Experimental group	Control group	t/χ^2^	P
Age (years old)	72.46±6.73	72.67±5.83	0.17	0.86
Female (cases %)	26 (52%)	30 (60%)	0.65	0.42
Uric acid (μmol/L)	457.85±23.14	455.39±22.68	0.54	0.60
BMI (kg/m^2^)	22.43±2.86	22.29±2.45	0.26	0.79
Medical history (yrs)	3.06±0.77	3.12±0.68	0.41	0.68
Systolic blood pressure (mmHg)	166.52±12.41	167.21±13.08	0.27	0.79
Diastolic blood pressure (mmHg)	96.81±12.75	96.38±12.10	0.17	0.86

p>0.05.

### Ethical Approval

This study was approved by the Medical Ethics Committee of Baoding No.1 Central Hospital (No.:2021035; date: May 27, 2021), and all subjects gave informed consent and signed an informed consent form.

### Inclusion criteria:

- Patients who met the diagnostic criteria for hyperuricemia^11^;
- Patients aged >65 years who were conscious, without mental disorders, and able to actively cooperate with the treatment and implementation of nursing programs;
- Patients who met the diagnostic criteria for hypertension at the same time^12^;
- Patients with complete clinical data.

### Exclusion criteria:

- Patients with severe organic diseases that cannot be satisfactorily corrected;
- Patients who had recently taken uric acid-lowering drugs or had undergone dialysis;
- Patients with severe mental or nervous system diseases, or with cognitive impairment who cannot cooperate with the study satisfactorily;
- Patients with underlying diseases such as cerebrovascular disease, coronary heart disease and diabetes.

The control group was given conventional nursing care, including health education, guiding patients to receive relevant treatment, giving conventional antihypertensive drugs, lowering uric acid, protecting renal function and other basic therapeutic treatments.

The experimental group adopted a comprehensive nursing intervention based on the treatment plan of the control group, as follows:

### Popularization of knowledge of hypertension and hyperuricemia

The nurses popularized relevant knowledge of hypertension and hyperuricemia to patients to help them actively prevent and treat the disease.

Diet care: For patients with hypertension, sodium intake should be controlled below 6g per day. For those with hyperuricemia, the intake of purine-rich foods should be strictly limited.

###  Psychological nursing

The nurses followed up on patients’ psychological status in time to help them regulate their mood to avoid patients’ bad emotions from affecting the treatment effect.

### Exercise nursing

The nurses helped the patients to make corresponding exercise plans according to their own conditions. Control patients’ weight and improve body function.

### Clinical medication nursing

The nurses should continue to remind patients to take rational medication according to regulations, and urge them to regularly monitor blood uric acid and blood pressure.

### Observation indicators:

Treatment compliance: including adherence to standardized medication, regular measurement of blood pressure and uric acid, adherence to diet, adherence to smoking and alcohol cessation, and adherence to aerobic exercise.

### Clinical efficacy

The differences in systolic blood pressure, diastolic blood pressure and uric acid levels were compared between the two groups.

### Quality of life

The Generic Quality of Life Inventory-74 (GQOLI-74) was used to assess the quality of life. The higher the score, the better the patient’s quality of life.^13^

Comparative analysis of patient satisfaction: The Patient Satisfaction Questionnaire Short Form (PSQ-18)^14^ was used for patients, relatively satisfied, satisfied, and not satisfied, with total satisfaction = (very satisfied + relatively satisfied + satisfied)/total number of cases x 100%.

### Follow up

All patients were followed up for two years. The proportion of patients in both groups who developed gout or renal insufficiency was recorded, and their long-term treatment outcomes were compared.

### Statistical analysis

All data in this study were statistically analyzed by SPSS 20.0 software, and measurement data were expressed as (*χ̅*±*S*). Two independent sample t-test was used for comparison between groups, paired t-test was used to analyze data within groups, and c^2^ test was used for the comparison of rates, the power of test / confidence interval is 95%. P<0.05 indicates a statistically significant difference.

## RESULTS

After the comprehensive nursing intervention, the number of cases of compliance behaviors in the experimental group was significantly higher than that in the control group (p<0.05) (Table-II). The systolic and diastolic blood pressure and uric acid levels were significantly lower in the experimental group compared to the control group after the intervention (p=0.00) (Table-III).

**Table-II:** Comparative analysis of the improvement of compliance behavior between the two groups before and after treatment (*χ̅*±*S*) n=50.

Indicators		Experimental group (number of cases)	Control group (number of cases)	χ^2^	P
Adherence to standardized medication	Before intervention	12	11	0.07	0.81
	After intervention*	45	24	20.62	0.00
Regular measurement of blood pressure and uric acid	Before intervention	8	13	1.51	0.22
	After intervention*	44	30	10.19	0.00
Adherence to diet	Before intervention	13	10	0.51	0.48
	After intervention*	40	27	8.73	0.00
Adherence to changing bad living habits	Before intervention	16	14	0.19	0.66
	After intervention*	43	33	5.48	0.02
Adherence to aerobic exercise	Before intervention	11	14	0.48	0.50
	After intervention*	47	38	6.35	0.01

p<0.05.

**Table-III:** Comparative analysis of the treatment effect between the two groups before and after the intervention (*χ̅*±*S*) n=50.

Indicators		Experimental group	Control group	t	p
Systolic blood pressure (mmHg)	Before intervention	166.52±12.41	167.21±13.08	0.27	0.79
	After intervention*	128.36±10.03	153.62±11.82	9.21	0.00
Diastolic blood pressure (mmHg)	Before intervention	96.81±12.75	96.38±12.10	0.17	0.86
	After intervention*	73.31±9.04	85.36±10.36	4.88	0.00
Serum uric acid (μmol/L)	Before intervention	457.85±23.14	455.39±22.68	0.54	0.60
	After intervention*	274.58±27.60	296.06±23.47	3.40	0.00

* p<0.05

The scores of physical function, psychological function, social function and material life status improved significantly in the experimental group compared to the control group after the intervention (p=0.00) (Table-IV). The satisfaction rate of the experimental group was 94%, which was significantly higher than 78% in the control group (p=0.02) (Table-V). The proportion of patients in the experimental group who developed gout was significantly lower than that in the control group (p=0.03) (Table-VI).

**Table-IV:** Comparative analysis of quality of life scores between the two groups before and after the intervention (*χ̅*±*S*) n=50.

Indicators		Experimental group	Control group	t	p
Physical function	Before intervention	42.31±5.38	42.40±5.41	0.44	0.56
	After intervention*	55.73±6.72	48.46±6.25	4.67	0.00
Psychological function	Before intervention	45.28±7.30	45.13±6.95	0.63	0.44
	After intervention*	56.26±6.47	51.52±6.81	3.87	0.00
Social function	Before intervention	59.68±9.57	60.08±9.27	0.51	0.48
	After intervention*	67.49±8.12	64.73±8.25	5.78	0.00
Material life status	Before intervention	47.07±8.76	47.12±8.18	0.73	0.34
	After intervention*	56.40±7.14	52.18±7.33	6.87	0.00

* p<0.05.

**Table-V:** Comparative analysis of satisfaction between the two groups (*χ̅*±*S*) n=50.

Group	Very satisfied	Relatively satisfied	Satisfied	Not satisfied	Total satisfaction*
Experimental group	27	16	4	3	47 (94%)
Control group	19	14	6	11	39 (78%)
c^2^					5.32
p					0.02

* p<0.05.

**Table-VI:** Comparative analysis of follow-up results between the two groups (*χ̅*±*S*) n=50.

Group	Gout	Renal insufficiency	Cardiovascular disease	Prevalence
Experimental group	2	0	0	2 (4%)
Control group	5	1	3	9 (18%)
c^2^				5.01
p				0.03

p<0.05.

## DISCUSSION

It was confirmed in our study that after the comprehensive nursing intervention, the number of cases of compliance behaviors in the experimental group was significantly higher than that in the control group. The systolic and diastolic blood pressure and uric acid levels were significantly lower in the experimental group compared to the control group after the intervention. The scores of physical function, psychological function, social function and material life status improved significantly in the experimental group compared to the control group after the intervention. The satisfaction rate of the experimental group was significantly higher than the control group.

Hyperuricemia (HU), a common disease in middle-aged and elderly patients, occurs as a result of a series of metabolic diseases which caused by high blood uric acid levels in the organism mainly due to disorders of purine metabolism.^15^ Hyperuricemia is the biochemical basis of gout. Some studies have pointed out that 5% to 19% of patients with hyperuricemia develop gout later.^16^ It has been shown in related studies that elevated levels of uric acid in the body have a strong correlation with hypertension, and HU has become a risk factor for causing cardiovascular disease.^17^ Data showed^18^ that HU often coexists with cardiovascular disease, hypertension, obesity, dyslipidemia, and diabetes.

HU has become an independent risk factor for cardiovascular disease. Despite the treatment methods for patients with hypertension and hyperuricemia are now relatively mature, their prognostic links are still weak. To explain the reasons, patients and their families are not sufficiently aware of the etiology of the disease, precautions, and reasonable lifestyle habits, which bring some interference in the prognosis of the disease.^19^ Therefore, health education is provided to patients during hospitalization to popularize the basic knowledge of the disease, establish reasonable dietary standards, perform an appropriate exercise, and adhere to medication as prescribed, etc., to help patients improve their knowledge of disease prevention and treatment. Comprehensive nursing interventions can be combined with the prognostic regression of the disease and the psychological characteristics of patients to take targeted nursing guidance, while continuous care after discharge can reduce adverse emotions, which is a scientific extension of in-hospital care.^20^

The proportion of patients in the experimental group who developed gout was significantly lower than that in the control group. It can be seen that comprehensive nursing intervention is of great clinical significance and can significantly improve clinical efficacy. Specifically, going through a comprehensive nursing intervention means that patients are able to receive targeted interventions at each stage, which is of great significance for improving treatment outcomes. The conclusion of this study provides additional clinical reference for the clinical application of comprehensive nursing intervention in hypertensive patients with HU.

### Limitations

It includes small sample size and the follow-up period was short. In future, more patients will be included and follow-up time will be extended to more objectively evaluate the pros and cons of this intervention, so as to benefit more patients.

## CONCLUSION

To put it in a nutshell, after comprehensive nursing intervention, the clinical efficacy and long-term therapeutic effect of HU patients with hypertension were significantly improved. Therefore, comprehensive nursing intervention plays a vital role in the treatment and prognosis of hypertension combined with HU.

### Authors’ Contributions:

**NL:** Carried out the studies, participated in collecting data, and drafted the manuscript, and are responsible and accountable for the accuracy or integrity of the work.

**JL:** Performed the statistical analysis and participated in its design.

**PC:** Participated in acquisition, analysis, or interpretation of data and draft the manuscript.

All authors read and approved the final manuscript.

## References

[1] Parmaksız E, Parmaksız ET (2022). Uric acid as a prognostic predictor in COVID-19. Pak J Med Sci.

[2] Yip K, Cohen RE, Pillinger MH (2020). Asymptomatic hyperuricemia:is it really asymptomatic?. Curr Opin Rheumatol.

[3] Jan MI, Khan RA, Sultan A, Ullah A, Ishtiaq A, Murtaza I (2019). Analysis of NT-proBNP and uric acid due to left ventricle hypertrophy in the patients of aortic valve disease. Pak J Med Sci.

[4] Petreski T, Ekart R, Hojs R, Bevc S (2020). Hyperuricemia, the heart, and the kidneys - to treat or not to treat?. Ren Fail.

[5] Eastwood J, Maitland-Scott I (2020). Patient Privacy and Integrated Care:The Multidisciplinary Health Care Team. Int J Integr Care.

[6] Chen MQ, Wang HY, Shi WR, Sun YX (2021). Estimate of prevalent hyperuricemia by systemic inflammation response index:results from a rural Chinese population. Postgrad Med.

[7] Dalbeth N, Lauterio TJ, Wolfe HR (2014). Mechanism of action of colchicine in the treatment of gout. Clin Ther.

[8] Ottaviani S, Molto A, Ea HK, Neveu S, Gill G, Brunier L (2013). Efficacy of anakinra in gouty arthritis:a retrospective study of 40 cases. Arthritis Res Ther.

[9] Coppell KJ, Abel SL, Freer T, Gray A, Sharp K, Norton JK (2017). The effectiveness of a primary care nursing-led dietary intervention for prediabetes:a mixed methods pilot study. BMC Fam Pract.

[10] Bardin T, Chales G, Pascart T, Flipo RM, Korng Ea H, Roujeau JC (2016). Risk of cutaneous adverse events with febuxostat treatment in patients with skin reaction to allopurinol. A retrospective, hospital-based study of 101 patients with consecutive allopurinol and febuxostat treatment. Joint Bone Spine.

[11] Su HY, Yang C, Liang D, Liu HF (2020). Research Advances in the Mechanisms of Hyperuricemia-Induced Renal Injury. Biomed Res Int.

[12] Lee JH, Kim KI, Cho MC (2019). Current status and therapeutic considerations of hypertension in the elderly. Korean J Intern Med.

[13] Wang Z, Cheng Y, Li J, Hu X (2021). Effect of integrated medical and nursing intervention model on quality of life and unhealthy emotion of patients with esophageal cancer undergoing radiotherapy. Am J Transl Res.

[14] Thayaparan AJ, Mahdi E (2013). The Patient Satisfaction Questionnaire Short Form (PSQ-18) as an adaptable, reliable, and validated tool for use in various settings. Med Educ Online.

[15] Mallat SG, Al Kattar S, Tanios BY, Jurjus A (2016). Hyperuricemia, Hypertension, and Chronic Kidney Disease:an Emerging Association. Curr Hypertens Rep.

[16] Waheed Y, Yang F, Sun D (2021). Role of asymptomatic hyperuricemia in the progression of chronic kidney disease and cardiovascular disease. Korean J Intern Med.

[17] Jayachandran M, Qu S (2021). Harnessing hyperuricemia to atherosclerosis and understanding its mechanistic dependence. Med Res Rev.

[18] Shen X, Wang C, Liang N, Liu Z, Li X, Zhu ZJ (2021). Serum Metabolomics Identifies Dysregulated Pathways and Potential Metabolic Biomarkers for Hyperuricemia and Gout. Arthritis Rheumatol.

[19] Vargas-Santos AB, Neogi T (2017). Management of Gout and Hyperuricemia in CKD. Am J Kidney Dis.

[20] Chandratre P, Mallen C, Richardson J, Muller S, Hider S, Rome K (2018). Health-related quality of life in gout in primary care: Baseline findings from a cohort study [published correction appears in Semin Arthritis Rheum. 2022;52:151800. Semin Arthritis Rheum.

